# 立体定向放疗治疗15例肺部小肿瘤患者的疗效分析

**DOI:** 10.3779/j.issn.1009-3419.2011.03.12

**Published:** 2011-03-20

**Authors:** 荣 余, 永恒 李, 广迎 朱

**Affiliations:** 100142 北京，北京大学临床肿瘤学院、北京肿瘤医院暨北京市肿瘤防治研究所放射治疗科，恶性肿瘤发病机制及转化研究教育部重点实验室 Key laboratory of Carcinogenesis and Translational Research (Ministry of Education), Department of Radiation Oncology, Peking University School of Oncology, Beijing Cancer Hospital and Institute, Beijing 100142, China

**Keywords:** 肺肿瘤, 放疗, 治疗结果, Lung neoplasms, Radiotherapy, Treatment outcome

## Abstract

**背景与目的:**

立体定向放疗在临床应用中取得了令人振奋的成果。本研究旨在评价直线加速器大分割立体定向放疗治疗肺部小肿瘤的可行性。

**方法:**

纳入2005年9月-2009年8月本院收治的15例符合要求的非小细胞肺癌（non-small cell lung cancer, NSCLC）及肺部转移瘤患者。放疗前在模拟机下测定肺部病灶的左右、前后、头尾三个方向的呼吸运动幅度，采用CT模拟定位，选择三维适形立体定向放疗。放疗剂量如下：5 Gy×10次/12天生物有效剂量[（biological effective dose, BED）=75 Gy]2例，6 Gy×8次/10天（BED=76.8 Gy）3例，8 Gy×6次/8天（BED=86.4 Gy）2例，12 Gy×4次/4天（BED=132 Gy）8例，病变直径1.5 cm-4 cm，毒性评价标准按照NCI-CTCAE 3.0版本进行。

**结果:**

15例患者患者近期疗效完全缓解（complete response, CR）率为60%，部分缓解（partial response, PR）率为20%，总有效率（CR+PR）为80%。1年局部控制率为100%，1年生存率为86.67%。肺的早期放射性反应0级2例，Ⅰ级9例，Ⅱ级4例，无Ⅲ级以上的副反应。

**结论:**

利用直线加速器对肺部小病灶实施48 Gy/4次/4天的照射是可行的。

## Abstract (en)

**Background and objective:**

It has been known that stereotactic radiotherapy has been widely used in clinical practice. The aim of this study is to evaluate the feasibility to treat small lung neoplasms with hypofraction stereotactic radiotherapy.

**Methods:**

From September 2005 to November 2009, 15 patients with non-small cell lung cancer and solitary metastatic lung cancer were treated with hypofration stereotactic radiotherapy. Dose volume histogram was used to evaluated treatment plans. Lesion diameter 1.5 cm-4.0 cm. Toxicity was evaluated with the NCI-CTCAE 3.0 version. A dose 48 Gy-50 Gy was given in 4-10 fractions.

**Results:**

The short-term results showed that the complete response (CR) rate, the partial response (PR) rate, the overall response (CR+PR) rate, the one-year local control rate, the one-year survival rate was 60%, 20%, 80%, 100% and 86.67% respectively. Two patients had radiation pneumonitis of 0 grade. Acute radiation pneumonitis of grade Ⅰ occurred in 9 patients, 4 patients had radiation pneumonitis of grade Ⅱ and no patient developed serious (grade Ⅲ) radiation pneumonitis.

**Conclusion:**

The hypofraction stereotactic radiotherapy is safe for small lung neoplasms.

随着精确定位技术、影像技术和聚焦式放疗技术的发展，立体定向放疗治疗肿瘤的研究和临床应用日益广泛。立体定向放疗可以提高每次分割照射剂量、减少分割次数，缩短总疗程时间，从而增加相对生物效应剂量，提高肿瘤局部控制率，尤其在治疗增殖较快的肿瘤时效果更加明显。Fowler等^[1]^发现非小细胞肺癌（non-small cell lung cancer, NSCLC）是一种增殖较快的肿瘤，倍增时间为3.0 d-3.5 d，而小细胞肺癌（small cell lung cancer, SCLC）较NSCLC增殖速度更快，总疗程时间延长会导致肿瘤局部控制率降低，生存率降低。根据以往的临床研究^[2]^，放疗在Ⅰ期-Ⅱ期NSCLC中主要应用于因内科疾病而无法接受手术或拒绝手术的患者。这主要由于经常规放疗后Ⅰ期-Ⅱ期患者的5年生存率仅为0-30%，明显低于手术切除所取得40%-80%的生存疗效。近几年来立体定向放疗在治疗早期NSCLC的临床应用研究十分活跃，其初步结果也令人振奋，较常规放疗相比立体定向放疗可明显降低治疗费用，提高局部控制率及总生存期^[3]^。临床Ⅰ期NSCLC的2年、3年局部控制率分别为95%^[4]^、78%^[5]^，5年生存率为70.8%^[6]^，与手术治疗相当。目前临床Ⅰ期NSCLC的手术治疗5年生存率为48%-61.7%^[7]^。本研究采用此技术治疗15例肺部小肿瘤，现将结果报告如下。

## 1 材料与方法

### 1.1 病例资料

2005年9月-2009年8月11例NSCLC及肺部转移瘤。病例选择标准：（1）病理确诊的NSCLC或原发肿瘤病理确诊并控制较好的肺内孤立转移病灶；（2）病灶为肺的外周；（3）病灶直径 < 4 cm；（4）无纵隔淋巴结转移；（5）无胸部放疗病史。根据当前的标准行组织病检，包括免疫组织化学。根据病理和/或临床诊断结果，分为肺腺癌（6例）、肺鳞癌（4例）和肺转移瘤（5例）。男12例，女3例；年龄49岁-81岁，平均年龄65岁。ECOG评分0分-1分。7例NSCLC患者因内科疾病无法耐受手术治疗，3例可手术的NSCLC患者拒绝手术，5例肺内转移癌患者不考虑手术治疗。详见表 1。

**1:** 15例肺部小肿瘤患者的临床资料 Clinical data of the 15 patients with small lung neoplasms

Clinical feature		*n*	%
Tumor size	≤4 cm	15	100.00
	> 4 cm	0	0
Gender	Male	12	80.00
	Female	3	20.00
Age	≤70	7	46.67
	> 70	8	53.33
Pathology	Adenocarcinoma	6	40.00
	Squamous cell carcinoma	4	26.67
	Metastatic carcinaoma	5	33.33
ECOG PS	0	4	26.67
	1	11	73.33
Reason for not surgery selected	Medical disease can not tolerated surgery	7	46.67
	Reject surgery	3	20.00
	Metastatic tumor	5	33.33

### 1.2 治疗方法

调强放疗采用Varian直线加速器，6兆伏-X线，适形放疗采用西门子Primus直线加速器，10兆伏-X线。先在模拟机室做固定体膜，采用仰卧位，双手抱肘置额上，体膜固定，并做好激光灯标记，在患者体表上标记皮肤与体膜、治疗床所处相对位置。定位CT扫描，层厚、层距均为5 mm。CT扫描图像经光缆传送至治疗计划系统，在肺窗勾画大体肿瘤体积（gross tumor volume, GTV）、根据呼吸动度外扩临床内在大体肿瘤体积（internal gross tumor volume, IGTV）及根据摆位误差外扩计划靶体积（plan tumor volume, PTV）。IGTV确定为GTV+呼吸动度，放疗前在模拟机下测定肺部病灶的左右、前后、头尾三个方向的呼吸运动幅度，上下外放5 mm-15 mm、左右外放3 mm-8 mm、前后外放3 mm-5 mm。PTV在IGTV基础上外放5 mm。由于考虑到大分割治疗单次剂量远高于常规分割，在相当于CTV的区域已有50 Gy以上的剂量，未单独勾画CTV。以PTV几何中心为射野等中心，采用4个-9个适形野，要求95%以上PTV体积接受处方剂量，要求V20≤15%，采用三维治疗计划系统制定治疗计划，放疗剂量：2例患者5 Gy×10次/12天生物有效剂量[（biological effective dose, BED）=75 Gy]，3例患者6 Gy×8次/10天（BED=76.8 Gy），2例患者8 Gy×6次/8天（BED=86.4 Gy），8例患者12 Gy×4次/4天（BED=132 Gy）。照射方法为每周5次。治疗机器为瓦里安内置600CD治疗系统或西门子primus治疗系统。

### 1.3 疗效和毒副反应评价

患者在治疗结束后，分别于治疗后两年之内每3个月复查胸部CT，按RECIST肿瘤近期疗效标准进行疗效评价。毒副反应评价参考NCI-CTCAE 3.0。

### 1.4 统计方法

患者随访从治疗结束开始，生存率和局部控制率从治疗开始日算起。应用SPSS 11.5软件包，采用*Kaplan-Meirer*法计算生存率。

## 2 结果

末次随访时间为2010年6月12日，随访率为100%，中位随访期为19.7个月。

### 2.1 近期疗效

全部患者近期疗效完全缓解（complete response, CR）率为60%（9/15），部分缓解（partial response, PR）率为20%（3/15），稳定（stable disease, SD）率为20%（3/15），进展（progresive disease, PD）0例。总有效率（CR+PR）为80%（12/15）。

### 2.2 肿瘤的局部控制率与生存情况

全部患者6个月局部控制率为100%，其中1例随访未到1年，余14例患者1年局部控制率均为100%。10例原发肺癌患者均生存1年以上，肺转移癌死亡2例。详见表 2。

**2:** 15例肺部小肿瘤患者的局部控制率和生存率 Local control rate and overall survival rate data of the 15 patients with small lung neoplasms

	*n*	6 months local control rate	1 year local control rate	1 year survival rate
Primary lung cancer	10	100%	100%	100%
Metastatic lung cancer	5	100%	100%	60%
Total	15	100%	100%^*^	86.67%
^*^1 case followed-up less than 1 year.				

### 2.3 毒副反应

一般反应（表现为乏力、纳差、恶心）3例，给予营养支持治疗后好转。血液系统的放射性损伤较轻，仅2例出现Ⅰ级反应，主要为白细胞减少和血小板减少。合并化疗者增加了食管毒性反应，化疗导致骨髓抑制应用升白药物后恢复良好。肺的放射反应0级2例，Ⅰ级9例，Ⅱ级4例。Ⅱ级的4例患者有咳嗽、胸部不适等症状，无发热，放射学上肺纤维化范围较大，不影响日常活动，治疗后症状消失。未发现Ⅲ级及以上放射性肺损伤。未发现有1级以上放射性食管炎。详见表 3。

**3:** 15例肺部小肿瘤患者放疗毒副反应 Radiation toxicity data of the 15 patients with small lung neoplasms

Toxicity	Grade 0	Grade Ⅰ	Grade Ⅱ	Grade Ⅲ	Grade Ⅳ
Radiation pneumonitis	2	9	4	0	0
Radiation esophagitis	15	0	0	0	0
Bone marrow suppression	13	2	0	0	0
Systemic reaction	12	3	0	0	0

## 3 讨论

在本研究采用立体定向快速放疗的方法治疗15例原发肺癌或肺转移癌患者，随访率为100%，中位随访期为19.7个月，全部患者近期疗效总有效率达80 %（12/15），1年局部控制率达100%。治疗效果较好，毒副反应轻微。本组患者多合并有心肺等基础疾病，大分割放疗只针对原发灶，不做纵隔区预防性放射治疗，且患者的病灶均 < 4 cm，治疗计划设计时控制全肺V20 < 15%，患者治疗中及结束后放射性肺和食管反应轻微，仅从随诊CT片上有小范围局部纤维变，而患者多数无症状，提示大分割精确放疗可作为肺部小肿瘤治疗的主要手段之一。

靶区勾画均由GTV+呼吸移动形成IGTV，并在此基础上外扩5 mm形成PTV照射，由于考虑到大分割治疗单次剂量远高于常规分割，在CTV的区域已有相当于常规放疗50 Gy左右的剂量，且从临床治疗观察大部分肿瘤治疗后未达CR，为更好的控制肿瘤本身，增加肿瘤处方剂量，减少周边正常组织损伤，未外放CTV。结果表明一年内未见肿瘤边缘的局部复发。

本组肺部小肿瘤患者以NSCLC所占比例最大。近几年来立体定向放射治疗早期NSCLC的临床应用研究十分活跃，其初步结果也令人振奋。Timmerman等^[8]^报道了59例不能手术的Ⅰ期NSCLC（44例T1、11例T2）接受立体定向放射治疗，54 Gy/3次/1-2周，3年的肿瘤局部控制率为90.6%，3年总生存率为55.8%，中位生存时间为48.1个月。Videtic等^[9]^报道26例不能手术的Ⅰ期NSCLC 3年的肿瘤局部控制率为94.4%，3年总生存率为52%。Onishi等^[10]^报道的日本13家医院采用立体定向高分次剂量治疗245例早期NSCLC的结果：临床Ⅰa期（T1N0M0）155例，Ⅰb期（T2N0M0）90例，分次剂量3 Gy-12 Gy，总剂量18 Gy-75 Gy，分1次-22次不等。BED≥100 Gy的173例，BED < 100 Gy的72例。3年、5年总生存率分别是56%和47%，3年、5年肿瘤特异生存率分别为78%和78%；BED≥100 Gy的生存率明显高于BED < 100 Gy。Nagata等^[11]^报道了45例接受单次大剂量放疗的Ⅰ期肺癌，单次剂量12 Gy，共4次，总疗程为5 d-13 d，平均随访时间为30个月，Ⅰa期1、3年总生存率为92%和83%，Ⅰb期1、3年总生存率为82%和72%。Ricardi等^[12]^报道了62例接受立体定向放疗的Ⅰ期NSCLC，剂量45 Gy分3次完成，总疗程1周-2周。3年的肿瘤局部控制率为87.8%，3年总生存率为57.1%，肿瘤特异生存率为72.5%。Onishi等^[6]^2007年再次报道日本14家医院高分次剂量治疗257例早期NSCLC的结果：BED≥100 Gy的5年总生存率为70.8%，明显高于BED < 100 Gy的5年总生存率30.2%。可见采用立体定向高分次剂量治疗早期NSCLC是一个有效的方法，局部控制率和生存率都远高于常规分割放疗。本组研究显示使用直线加速器采用快速高分次剂量治疗技术治疗肺部小肿瘤安全、有效，且采用48 Gy/4次/4天（BED=132 Gy）分割方式治疗无严重毒副反应。

放射损伤是影响早期NSCLC放疗疗效的重要因素，而三维适形立体定向大分割放射治疗的急性肺损伤并不增加，还有降低可能约为0-4%^[13-17]^，Ⅲ级以上放射性肺炎发生率为2.3%-3.3%^[16, 18, 19]^，Ⅲ级放射性食管炎为0.8%^[20]^。Onishi等^[6]^报道的日本14家医院采用立体定向高分次剂量治疗257例早期NSCLC的结果显示：Ⅱ级以上的放射性肺炎发生率为5.4%。Zimmermann等^[18, 19]^报道的立体定向放射治疗伴有内科疾病而不能手术的30例Ⅰ期NSCLC的结果显示：仅1例发生Ⅲ级急性放射性肺损伤，发生率为3%，无Ⅲ级以上放射性损伤发生。Nagata等^[11]^报道的45例接受单次大剂量放疗的Ⅰ期肺癌，未发现Ⅲ级及Ⅲ级以上放射性副反应。Fakiris等^[21]^报道的70例接受单次大剂量放疗的Ⅰ期NSCLC未发现放疗相关的严重副反应。本组病例年龄大多超过65岁，合并有心肺疾病，靶区较小，V20控制在15%以下，未行化疗，放射性肺损伤较轻，局限于Ⅰ级-Ⅱ级放射性肺炎，发生率86.67%。13例患者胸部CT提示存在放射性改变，其中4例患者有咳嗽、胸部不适等症状，无发热、憋气，不影响日常活动，治疗后症状消失。随访3个月-6个月后胸部CT大多有靶区内小范围纤维化改变，患者无自觉症状。

本文初步研究结果提示使用直线加速器采用大分割放射治疗技术治疗肺部小肿瘤是安全、有效的，近期疗效满意，是早期肺癌者可首选的手段之一。但本研究病例数有限，随访期尚短，疗效及晚反应损伤的评估有待进一步观察和研究。
